# High IKZF1/3 protein expression is a favorable prognostic factor for survival of relapsed/refractory multiple myeloma patients treated with lenalidomide

**DOI:** 10.1186/s13045-016-0354-2

**Published:** 2016-11-21

**Authors:** Maryam Pourabdollah, Mohammad Bahmanyar, Eshetu G. Atenafu, Donna Reece, Jian Hou, Hong Chang

**Affiliations:** 1 Department of Laboratory Medicine and Pathobiology, University of Toronto, Toronto, Canada; 2 Department of Biostatistics, University of Toronto, Toronto, Canada; 3 Department of Hematology and Medical Oncology, University of Toronto, Toronto, Canada; 4 Department of Hematology, Shanghai Chang Zheng Hospital, Shanghai, China; 5 Department of Laboratory Hematology, University Health Network, 200 Elizabeth Street, 11E-413, Toronto, ON M5G 2C4 Canada

## Abstract

The aim of this study is to assess nucleoprotein expression of IKZF1/3 in patients with relapsed/refractory multiple myeloma (MM) who received lenalidomide-based therapy and correlated them with their clinical outcomes. A total of 50 patients diagnosed with MM were entered in the study with the median follow-up of 86.4 months. By immunohistochemistry (IHC), IKZF1 and IKZF3 were expressed in 72 and 58% of the cases, respectively. IKZF1 and IKZF3 expressions were associated with longer median progression free survival (*P* = 0.0029 and *P* < 0.0001) and overall survival (*P* = 0.0014 and *P* < 0.0001). IKZF3 expression also appears predicted a favorable response to the lenalidomide-based therapy.

**Electronic supplementary material:**

The online version of this article (doi:10.1186/s13045-016-0354-2) contains supplementary material, which is available to authorized users.

It has been demonstrated that lenalidomide causes selective degradation of IKZF1 (ikaros) and IKZF3 (aiolos) which are two essential transcription factors for myeloma cell proliferation [1, 2]. This anti-proliferative effect is mediated by downregulation of c-Myc and interferon regulatory factor 4 (IRF4) [3]. In particular, IKZF3 regulates expression of IRF4 which is linked with lenalidomide activity [4–6]. However, the clinical relevance of IKZF1/IKZF3 expressions in myeloma patients has not been established. Thus, we examined nuclear expression of IKZF1/3 and its correlation with clinical outcomes in patients with relapsed/refractory MM who received lenalidomide therapy.

A total of 50 patients diagnosed with MM in our institution were entered in the study. All had received lenalidomide-based therapy (lenalidomide plus dexamethasone) after relapse. The median follow-up after diagnosis was 7.2 years. The relevant clinical and laboratory features are summarized in Table 1.

**Table 1:** Clinical and laboratory features of MM patients treated with lenalidomide

Clinical feature	Total (*n* = 50)	IKZF1 high expression (*n* = 36)	IKZF1 low expression (*n* = 14)	*P* value	IKZF3 high expression (*n* = 29)	IKZF3 low expression (*n* = 21)	*P* value
Sex (M/F)	31/19	23/13	8/6	0.6590	19/10	12/9	0.5471
Age (year), median (range)	59(41–75)	57 (41–73)	59 (45–75)	0.6416	57 (41–69)	59 (44–75)	0.3105
International staging system, no. (%)				0.1785			0.1448
I	24 (48)	14 (38.89)	10 (71.43)		11 (37.93)	13 (61.90)	
Il	18 (36)	14 (38.89)	4 (28.57)		11 (37.93)	7 (33.33)	
III	5 (10)	5 (13.89)	0 (0)		5 (17.24)	0 (0)	
NA	3 (6)	3 (8.33)	0 (0)		2 (6.90)	1 (4.76)	
Hemoglobin concentration (g/L), median (range)	105 (76–147)	106 (76–147)	103 (86–132)	0.6027	104 (76–147)	107 (85–141)	0.3121
Calcium (mmol/L), median (range)	2.25 (1.98–2.57)	2.26 (1.98–2.57)	2.23 (2–2.55)	0.8162	2.25 (1.98–2.57)	2.25 (2–2.55)	0.7160
Creatinine (μmol/L), median (range)	76 (32–360)	86 (40–360)	67.58 (32–126)	0.0569	88 (57–360)	66 (32–126)	0.0072
Having lytic lesions, number of patients (%)	27 (54)	18 (50)	9 (64.29)	0.3628	12 (41.38)	15 (71.43)	0.0354
B2-microglobulin (mg/L)	3.08 (0.51–16.76)	3.50 (0.51–16.76)	2.96 (1.35–5.16)	0.5817	3.62 (0.51–16.76)	2.82 (1.07–5.16)	0.1293
Albumin (gr/L)	40.5 (28–47)	41 (28–47)	39 (29–44)	0.6151	41 (28–47)	40 (29–44)	0.9078
Prior therapies, no. (%)							
≥3	23 (46)	15 (41.67)	8 (57.14)	0.3242	12 (41.38)	11 (52.38)	0.4411
Thalidomide	29 (58)	22 (61.11)	7 (50)	0.4748	15 (51.72)	14 (66.67)	0.2907
Bortezomib	21 (42)	14 (38.89)	7 (50)	0.4748	12 (41.38)	9(42.86)	0.9168
ASCT	40 (80)	31 (86.11)	9 (64.29)	0.1180	25 (86.21)	15 (71.43)	0.2859
Response to lenalidomide plus dexamethasone, no. (%)							
Responsive	41 (82)	32 (88.89)	9 (64.29)	0.094	28 (96.55)	13 (61.90)	0.0025
Non-responsive	9 (18)	4 (11.11)	5 (35.71)		1 (3.45)	8 (38.1)	
Cytogenetics, no. (%)							
del (13q)				1.0000			0.7243
Positive	13 (26)	9 (25)	4 (28.57)		7 (24.14)	6 (28.57)	
Negative	37 (74)	27 (75)	10 (71.43)		22 (75.86)	15 (71.43)	
del (17p)				0.1966			0.2552
Positive	8 (16)	4 (11.11)	4 (28.57)		3 (10.34)	5 (23.81)	
Negative	42 (84)	32 (89)	10 (71.43)		26 (89.66)	16 (76.19)	
t(4;14)				1.0000			0.7163
Positive	9 (18)	7 (19.44)	2 (14.29)		6 (20.69)	3 (14.29)	
Negative	41 (82)	29 (80.56)	12 (85.71)		23 (79.31)	18(85.71)	
amp(1q21)				0.1228			0.1283
Positive	20 (40)	12 (33.33)	8 (57.14)		9 (31.03)	11 (52.38)	
Negative	30 (60)	24 (66.67)	6 (42.86)		20(68.97)	10 (47.62)	

CD138 and IKZF1/3 immunohistochemical (IHC) staining were performed on the bone marrow aspiration/biopsy specimens taken before starting lenalidomide. CD138 positive myeloma cell aggregates (Additional file 1: Figure S1B) were examined for IKZF1/3 expression (Additional file 1: Figure S1C, D). H-score method (range 0–300) according to staining intensity and percentage of myeloma cells was applied. The median H-scores for IKZF1 and IKZF3 were 150 and 200, respectively, and high or low expression was based on above or below the median H-score (Fig. 1a).

**Fig. 1:**
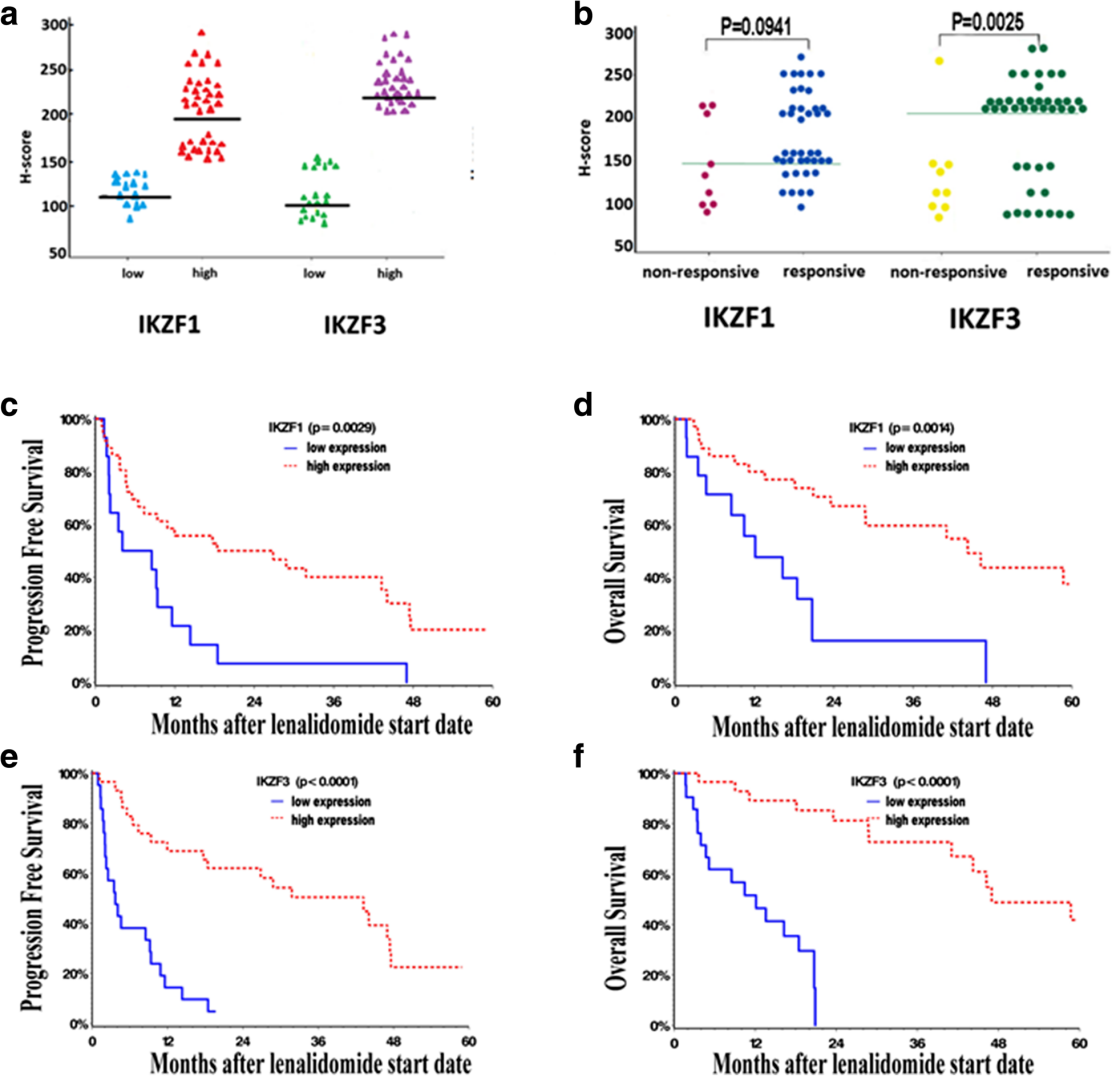
Expression of IKZF1 and IKZF3 proteins in tumor cells, measured by H-score (**a**) and their correlation with clinical response (**b**). Progression free survival (PFS) and overall survival (OS) in relation to IKZF1 nuclear expression (**c**, **d**), and in relation to IKZF3 nuclear expression (**e**, **f**) detected by IHC, respectively

Of the 50 MM cases, IKZF1 and IKZF3 were expressed in 36 (72%) and 29 (58%) cases, respectively. Twenty-eight of 29 cases (97%) with high IKZF3 expression also showed IKZF1 positivity (*P* < 0.0001). High IKZF3 (*P* = 0.0025), but not IKZF1 expression (*P* = 0.094) was strongly correlated with clinical response (Fig. 1b). Patients with high IKZF1 or IKZF3 expression showed longer PFS (median 22.6 vs. 6.3 months, *P* = 0.0029; or 43.2 vs. 3.7 months; *P* < 0.0001) and OS (median 44.3 vs. 12.1 months, *P* = 0.0014; or 47 vs. 12.1 months; *P* < 0.0001) (Fig. 1c–f), respectively. In addition, the group with both high IKZF1/3 expression was associated with longer PFS (median 31.8 vs. 3.9 months, *P* < 0.0001) and OS (median OS 58.7 vs. 12.1 months, *P* < 0.0001), whereas the group with both low expression was correlated with shorter PFS (median 4.1 vs. 26.8 months, *P* = 0.0003) and OS (median 12.1 vs. 46.2 months, *P* = 0.0002). Of note, high IKZF3 expression appeared associated with higher creatinine but with less lytic lesion, and by multivariable analysis, high IKZF3 expression remained an independent poor risk factor for PFS (*P* < 0.0001) and OS (*P* < 0.0001) after adjusting these two covariates. There was no significant association between IKZF1/3 protein expression and other clinical or biological risk factors (Table 1).

Previous studies have indicated controversial results about the relationship between Ikaros expression level and resistance to lenalidomide. Lu et al. [7] found that some MM cell lines with higher expressions of IKZF1 or IKZF3 showed resistance to the drug; in contrast, Zhu et al. [8] showed that low IKZF1 transcript levels were correlated with poor response to IMiDs. They also found that higher IKZF1 but not IKZF3 gene expression was associated with better OS. Our study demonstrates that expression of IKZF1/3 proteins (especially IKZF3) is correlated with better outcome in refractory MM patients treated with lenalidomide. A possible explanation for this observation is that in the presence of high IKZF1/3 levels, myeloma cells are more dependent on IKZF-associated signaling for proliferation. Particularly, IKZF3 is linked to plasma cell development and lenalidomide efficacy as IKZF3 is specifically required for the generation of long-lived plasma cells and it has been shown to be reduced by lenalidomide [9, 10].

To the best of our knowledge, this is the first report to show a correlation between IKZF1/3 protein expressions and clinical outcomes in refractory MM treated with lenalidomide. However, this study has limitations as it is retrospective with limited sample size. Nevertheless, as paraffin IHC is routinely available, robust, and inexpensive, if confirmed in a larger prospective study, IKZF1/3 (especially IKZF3) immunostaining can be readily adopted in clinical practice for prediction of drug response and clinical outcomes in MM patients receiving lenalidomide therapy.

## Additional file

Additional file 1: Figure S1. Bone marrow biopsy from a patient with MM. (A) H&E stain, (B) CD138 for myeloma cells, (C) Nuclear expression of IKZF1, (D) Nuclear expression of IKZF3.
