# Supplementary material for: High IKZF1/3 protein expression is a favorable prognostic factor for survival of relapsed/refractory multiple myeloma patients treated with lenalidomide
Source: J Hematol Oncol. 2016 Nov 21;9:123. doi: 10.1186/s13045-016-0354-2 (PMC5120536; doi:10.1186/s13045-016-0354-2)
Supplement: Additional file 1: Figure S1. — Bone marrow biopsy from a patient with MM. (A) H&E stain, (B) CD138 for myeloma cells, (C) Nuclear expression of IKZF1, (D) Nuclear expression of IKZF3. [file 13045_2016_354_MOESM1_ESM.docx]

# Figure 1. Bone marrow biopsy from a patient with MM. (A): H&E stain, (B): CD138 for myeloma cells, (C): Nuclear expression of IKZF1, (D): Nuclear expression of IKZF3.

# (A) (B )

**
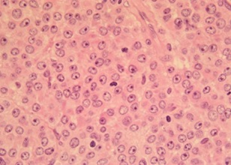

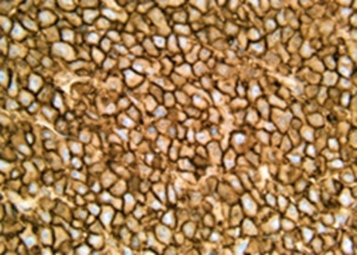
**

**(C) (D)**

**
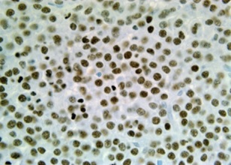

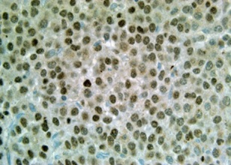
**
